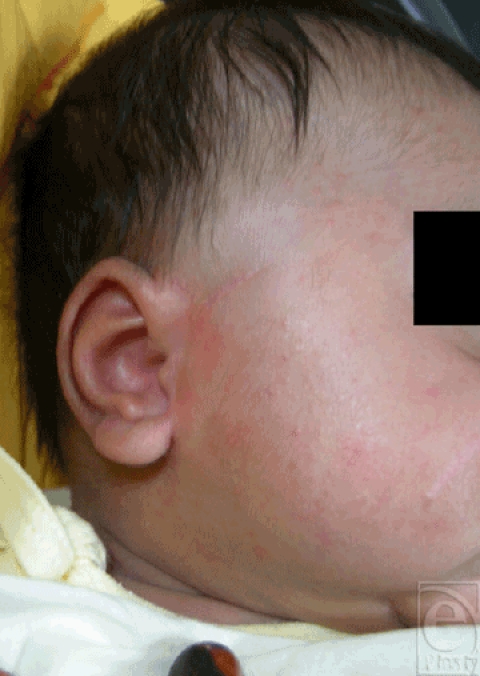# Facial Laceration at Caesarean Section: Experience With Tissue Adhesive

**Published:** 2009-01-09

**Authors:** Sanjay Saraf

**Affiliations:** Department of Plastic Surgery, NMC Specialty Hospital, Dubai, United Arab Emirates

## Abstract

**Background:** The fetal laceration is one of the most commonly identified injuries at the caesarean delivery. The incidence in the literature has been reported to be as high as 3%. The management of such injuries has remained a perplexing problem for both the physician and the parents. **Materials and Methods:** We present a case of a newborn who accidentally sustained laceration over the face during a caesarean delivery. A review of the literature and management of lacerations with tissue adhesives has been presented. **Results:** The laceration was successfully managed with tissue adhesive alone with good aesthetic outcome. **Conclusion:** Topical 2-octylcyanoacrylate tissue adhesives can be an effective alternative therapy for traditional devices for closing simple low-tension lacerations.

Caesarean section is the most common major surgical procedure among women of reproductive age and the incidence of accidental fetal laceration during caesarean is not rare, especially when it is performed for emergency indications. The incidence in the literature has been reported to be around 0.7% to 3%.^1^–^3^ The management of such lacerations in newborns always poses a unique challenge to the surgeon in terms of offering uncomplicated procedure in minimal time with the best possible aesthetic outcome.

Tissue adhesives have lately been introduced as an alternative to standard wound closure for repairing simple traumatic low-tension lacerations. They form a strong bond across apposed wound edges, allowing normal healing to occur below. They offer the benefit of decreased procedure time, less pain, and comparable aesthetic outcome to the standard wound closure in carefully selected patients. The judicious use and understanding of limitations and technical aspects specific to tissue adhesives will ensure optimal results.

## CASE SUMMARY

**Figure 1:**
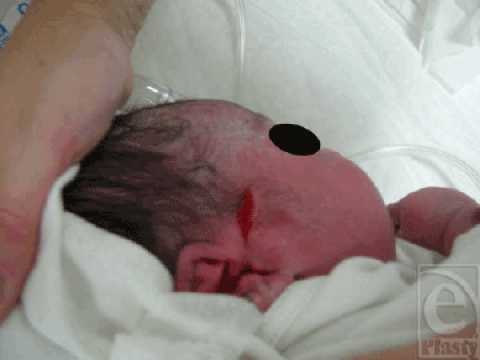
The female newborn, weighing 3.25 kg, accidentally sustained a laceration over the right side of the face and temporal region.

A full-term, 36-week primipara underwent caesarean delivery for fetal distress in emergency department. The female newborn, weighing 3.25 kg, accidentally sustained a laceration over the right side of the face and temporal region (Fig 1). The Apgar scores at 1 and 5 minutes were 6 and 7, respectively. The neonate was referred to us for wound management. In view of the low Apgar scores and reluctancy of the parents for subjecting the newborn to surgery, the wound was primarily closed with a 2-octylcyanoacrylate tissue adhesive (Dermabond, Ethicon Inc, Somerville, NJ) with satisfactory aesthetic outcome (Fig 2).

## DISCUSSION

Tissue adhesives are being increasingly used to replace sutures, staples, and adhesive strips in the management of surgical and traumatic wounds. Several clinical studies have found tissue adhesives as an effective alternative to standard wound closure techniques. The studies reported no significant differences between tissue adhesives and standard wound closure techniques in short- or long-term aesthetic outcomes.^4^–^11^

Although many types of tissue adhesives are available in market, 2-octylcyanoacrylate^12^ is presently the only tissue adhesive approved by Food and Drug Administration for closure of incised skin. In addition to the skin closure, 2-octylcyanoacrylate has also been found to be effective in protecting wounds from external bacterial invasion.^13^,^14^

They form a strong bond across apposed wound edges, allowing normal healing to occur below. They offer many potential advantages over standard wound closure, including decreased procedure time, less pain, and low-cost alternative with comparable cosmetic outcome to the standard wound closure in simple traumatic low-tension lacerations.

Although tissue adhesives have many advantages, understanding of the indications, contraindications, and a proper method of application is important for achieving the desired results.

The main indications are simple low-tension wounds and surgical wounds in areas of high cosmetic significance where good dermal approximation has already been achieved. They are often useful for pediatric wounds where suture removal can be difficult and for lacerations sustained during sports needing immediate attention.

The contraindications are high-tension wounds, mucosal or wet wounds, wounds with evidence of active infection, wounds regularly exposed to body fluids, wounds over dense hair-bearing area or skin precoated with petroleum jelly, known hypersensitivity to cyanoacrylate or formaldehyde, and an open wound where the polymerized material can elicit a foreign body reaction.

Care should be taken in application to areas near the eyes because of the possibility of runoff and in mid-facial wounds where underlying vital structures injury especially facial nerve needs to be excluded prior to the application.

It is important to understand that these topical agents are usually effective in low-tension wounds and can be used alone but in high-tension wounds, in addition, deep sutures are recommended. If not used properly, and tissue adhesive spills into the wound, the adhesive acts as a barrier to epithelialization and may retard healing. The adhesive also has the potential to cause a foreign-body reaction and increased risk of infection. The proper eversion of the edges is extremely important for the successful closure with tissue adhesives.

In newborns, like in this case, as far as the satisfactory aesthetic result is concerned, it is probable that wound healing may be slightly different or even better than healing in the normal adult. The studies have shown that wound healing in the fetus is fundamentally different from healing in the adult. The fetus has the ability to heal skin injury without the scarring, inflammation, and contraction that often accompany adult wounds. Fetal wounds heal instead with regeneration of epithelial and mesenchymal tissues and restoration of normal skin architecture.^15^ The profiles of fetal proteoglycans, collagens, and growth factors are different from those in adult wounds. The less-differentiated state of fetal skin is probably an important characteristic responsible for scarless repair.^16^

This difference between fetal and adult skin wound healing appears to reflect processes intrinsic to fetal tissue, such as the unique fetal fibroblasts, a more rapid and ordered deposition and turnover of tissue components, extracellular matrix, and particularly, a markedly reduced inflammatory infiltrate and cytokine profile.^16^,^17^

## SUMMARY

Suturing of the wound remains a standard wound closure technique; however, topical 2-octylcyanoacrylate tissue adhesives can be an effective alternative therapy for traditional devices for closing simple low-tension lacerations. The proper assessment of the wound, especially wound tension and careful application of the adhesive with proper wound eversion, is a must to achieve satisfactory outcome.

## Figures and Tables

**Figure 2:**